# Investigating Optimal Autologous Cellular Platforms for Prenatal or Perinatal Factor VIII Delivery to Treat Hemophilia A

**DOI:** 10.3389/fcell.2021.678117

**Published:** 2021-08-10

**Authors:** Christopher Stem, Christopher Rodman, Ritu M. Ramamurthy, Sunil George, Diane Meares, Andrew Farland, Anthony Atala, Christopher B. Doering, H. Trent Spencer, Christopher D. Porada, Graça Almeida-Porada

**Affiliations:** ^1^Wake Forest Institute for Regenerative Medicine, Fetal Research and Therapy Program, Wake Forest School of Medicine, Winston-Salem, NC, United States; ^2^Special Hematology Laboratory, Wake Forest Baptist Medical Center, Wake Forest School of Medicine, Winston-Salem, NC, United States; ^3^Department of Pediatrics, Aflac Cancer and Blood Disorders Center, Emory University School of Medicine, Atlanta, GA, United States

**Keywords:** gene therapy, hemophilia A, FVIII, endothelial progenitor cell, mesenchymal stromal (stem) cell therapy

## Abstract

Patients with the severe form of hemophilia A (HA) present with a severe phenotype, and can suffer from life-threatening, spontaneous hemorrhaging. While prophylactic FVIII infusions have revolutionized the clinical management of HA, this treatment is short-lived, expensive, and it is not available to many A patients worldwide. In the present study, we evaluated a panel of readily available cell types for their suitability as cellular vehicles to deliver long-lasting FVIII replacement following transduction with a retroviral vector encoding a B domain-deleted human F8 transgene. Given the immune hurdles that currently plague factor replacement therapy, we focused our investigation on cell types that we deemed to be most relevant to either prenatal or very early postnatal treatment and that could, ideally, be autologously derived. Our findings identify several promising candidates for use as cell-based FVIII delivery vehicles and lay the groundwork for future mechanistic studies to delineate bottlenecks to efficient production and secretion of FVIII following genetic-modification.

## Introduction

Hemophilia A (HA) is an X-linked coagulopathy that results from deficiency in functional coagulation factor VIII (FVIII) in circulation (Plug et al., 2006; High, 2012; Gilbert et al., 2015). Current treatments consist of frequent FVIII infusions that are expensive, are unavailable to ∼75% of HA patients, and cannot guarantee lifelong disease management, as up to 30% of patients develop inhibitors that render treatment ineffective, causing significant morbidity and mortality (Kaveri et al., 2007). There is thus an urgent need for more effective, readily available, affordable treatments that avoid these immunological hurdles and provide long-lasting correction. Ongoing HA clinical trials using adeno-associated virus (AAV) vectors to deliver the fVIII transgene have shown promise, with some patients achieving factor-independence for >2 years (Rangarajan et al., 2017; Chapin and Monahan, 2018). However, FVIII levels are dropping at the 3-year timepoint, suggesting this approach may not yield lifelong correction. Additionally, AAV-induced transaminase elevation and anti-capsid immunity have obliged prophylactic steroids in all subsequent patients. Furthermore, pre-existing immunity to AAV in up to 60% of individuals limits who could benefit from this therapy (Calcedo et al., 2011; Li et al., 2012; Calcedo and Wilson, 2013), and even a single exposure of “AAV-naïve” patients immunizes against the capsid, precluding follow-up dosing, and if needed (Fitzpatrick et al., 2018).

Approximately, 75% of HA patients have a family history, and fetal DNA within maternal blood enables non-invasive HA diagnosis as early as 7 gestational weeks (Tsui et al., 2011; Hudecova et al., 2017; Ragni, 2017; Camunas-Soler et al., 2018), making prenatal therapy possible (Porada et al., 2014). Adding to the advantages of prenatal correction are studies showing that fetal exposure to foreign antigen induces lifelong tolerance that resists postnatal challenge (Tran et al., 2001; Waddington et al., 2004; Colletti et al., 2008; Porada and Almeida-Porada, 2012; Porada et al., 2013; Peranteau et al., 2015; Takahashi et al., 2015; Davey et al., 2017). As such, even if not curative, prenatal treatment would permit postnatal therapies without fear of immune-related complications.

Both maternal and fetal safety are paramount for any prenatal treatment. As such, directly injecting viral vectors is not likely to initially be viewed as clinically viable, due to concerns over the possibility of altering the nascent germline and/or insertional mutagenesis (Almeida-Porada et al., 2019). Decades of research and over 50 clinical transplants (Loukogeorgakis and Flake, 2014; Almeida-Porada et al., 2016; McClain and Flake, 2016) have collectively demonstrated the safety and vast potential of cell-based prenatal therapies. We therefore reasoned that using cells as vehicles to carry the fVIII transgene would represent a safe, clinically acceptable approach to correct HA prenatally (Porada and Almeida-Porada, 2012; Porada et al., 2014).

Several studies sought to boost FVIII expression by engineering the fVIII transgene to include domains that enhance FVIII expression/secretion (Doering et al., 2002; Ide et al., 2007; Dooriss et al., 2009; Spencer et al., 2011) and by optimizing the vector construct used for delivery (Johnston et al., 2013, 2014). Herein, we focused on a less-explored issue: the cell used to deliver FVIII. Since FVIII is a large protein requiring complex post-translational modification (Mazurkiewicz-Pisarek et al., 2016), its expression can stress the endoplasmic reticulum, and not all cells possess the machinery needed to create fully functional FVIII protein (Pipe, 2005; Malhotra et al., 2008; Brown et al., 2011; Hennet, 2012; Lange et al., 2016; Zolotukhin et al., 2016). We therefore evaluated a panel of candidate cell types for their ability to be transduced with a retroviral vector encoding a B domain-deleted human fVIII transgene (HSQ) and subsequently express and secrete active FVIII, focusing on cell types most relevant to either prenatal or very early postnatal treatment, that can be autologously derived.

To perform prenatal autologous cell-based HA treatment requires a cell type that can safely be obtained from the fetus, gene-modified to constitutively express FVIII, expanded sufficiently *in vitro*, and returned to the fetus early enough in gestation to exploit the immunological advantages of prenatal intervention. For this reason, the first cells we considered were amniotic fluid-derived mesenchymal stromal cells (AF-MSC), which fulfill all of these criteria, can safely be obtained via amniocentesis, and are currently being explored for treating multiple diseases (De Coppi et al., 2007; Steigman and Fauza, 2007; Antonucci et al., 2011; Bollini et al., 2011a,b; Moorefield et al., 2011; Poloni et al., 2011; Shaw et al., 2011a,b; Fernandes et al., 2012; Murphy and Atala, 2013; Yang et al., 2013; Brown et al., 2014; Ramachandra et al., 2014; Zani et al., 2014; Chang et al., 2015; Li et al., 2015; Mariotti et al., 2015; Lazzarini et al., 2016; Balbi et al., 2017; Kunisaki, 2018; Subramaniam et al., 2018).

Despite the advantages of prenatal intervention, roughly 16,000 people within the US are already living with HA and could thus not benefit from prenatal therapy. Additionally, three scenarios would result in new HA patients not receiving prenatal treatment: (1) a family history of HA prompts parents to undergo fetal diagnosis, but they decline intervention; (2) a family history exists, but parents opt against fetal diagnosis/therapy; and (3) no family history exists, as over 25% of HA mutations arise *de novo*. Each of these three scenarios presents a different clinical situation with respect to the *autologous* cell types available to deliver FVIII. Parental consent to fetal diagnosis provides the most options. AF-MSC are available, and since the child is known to be affected, the parents could also collect the umbilical cord (UC) at birth, making it possible to isolate MSC (Islam et al., 2019; Selich et al., 2019) and/or endothelial progenitor cells (EPC) from the UC. The endogenous production of vWF makes EPC ideal for delivering FVIII, since binding to vWF stabilizes FVIII, prolongs its half-life (Rosenberg et al., 1998; Shi et al., 2010; Terraube et al., 2010), and reduces its immunogenicity (Kaveri et al., 2007; Lacroix-Desmazes et al., 2008). Lastly, MSC could also be isolated from bone marrow (BM) during early life, under prophylactic factor coverage. If the parents elect against fetal diagnosis, AF-MSC are not an option, but the other choices still exist. When no family history exists, diagnosis would only be made during the first 8–12 months of life, limiting the options to BM-derived MSC. Having considered these factors, we compared MSC from AF, UC, and BM and EPC from UC for their ability to serve as cellular vehicles for delivering a fVIII transgene during fetal or early postnatal life to correct HA.

## Materials and Methods

### Isolation and Culture of Human BM-MSC

Bone marrow mononuclear cells from two different biological donors were purchased from AllCells (Alameda, CA, United States) and their purchase approved by the Office of Human Research Protection at Wake Forest Health Sciences. Stro-1^+^/CD45^–^ BM-MSC were isolated via magnetic sorting (MACS Miltenyi Biotec, San Diego, CA, United States) and cultured on gelatin-coated flasks with Mesenchymal Stem Cell Growth Media (MSCGM) (Lonza, Walkersville, MD, United States) in a humidified 37°C incubator at 5% CO_2_ as previously described (Chamberlain et al., 2007; Sanada et al., 2013). BM-MSC were expanded and subsequently characterized by morphology and flow cytometry.

### Isolation and Culture of UCT-MSC and UCT-EPC From Human Umbilical Cord

Two different biological human cord tissue samples were kindly provided by Geoffrey O’Neill at CorCell (Las Vegas, NV, United States) and approved by the Office of Human Research Protection at Wake Forest Health Sciences. The samples were dissected into 3–5 cm segments. Blood vessels were carefully removed from tissues. Tissues were rinsed in Phosphate Buffered Saline (PBS) (Gibco, Grand Island, NY, United States) to remove any residual blood. Cord tissue was then digested in 300 units/ml collagenase type I (Sigma-Aldrich, St. Louis, MO, United States) in Hank’s Balanced Salt Solution (HBSS) with calcium and magnesium, for 1 h at 37°C on a shaker. After digestion, tissues were further minced with sterile surgical blades. Upon inhibition of tryptic enzyme activity by 10% fetal bovine serum (FBS) (Gibco, Grand Island, NY, United States), digested tissue was filtered over a 100 μm nylon mesh filter (Thermo Fisher Scientific, Rochester, NY, United States). Cells were washed, counted and split into two different gelatin-coated (Sigma-Aldrich, St. Louis, MO, United States) tissue culture-treated flasks. Cells in one flask were cultured in mesenchymal stem cell growth medium (MSCGM; Lonza, Walkersville, MD, United States), while cells in the other flask were grown in Endothelial Basal Media supplemented with cytokines, growth factors, and 10% FBS (EGM-2; Lonza, Walkersville, MD, United States). Media was changed after 72 h and subsequently every 3 days until cells reached 70% confluence.

### Culture of AF-MSC

Two AF-MSC lines were received from the Manufacturing Development Center (MDC) at the Wake Forest Institute for Regenerative Medicine, Winston-Salem, NC, United States and cultured in Chang’s Media as previously described as separate biological replicates (De Coppi et al., 2007). The cells were obtained after informed consent according to guidelines from the Office of Human Research Protection at Wake Forest Health Sciences.

### Characterization of UCT-MSC and UCT-EPC

Cells from the digested cord tissue and cultured in MSCGM were characterized as UCT-MSC based upon morphology and phenotype determined by flow cytometry as separate biological replicates. Antibodies used for phenotypic characterization included CD31, CD14, CD90, CD44, CD83, CD105, CD45, UEA-1, Stro-1, ICAM-1, CD146, CD11b, CD29, CD80, CD117, CD166, CD34, and CD309 (Becton Dickinson, Franklin Lakes, NJ, United States). UEA-1 was purchased from Sigma-Aldrich (St. Louis, MO, United States) and CD51 was purchased from Immunotec (Beckman-Coulter, Miami, FL, United States).

To obtain cord tissue-derived endothelial progenitor cells (UCT-EPC), cells from the digested cord tissue that had been cultured in EGM-2 were labeled with FITC-conjugated Ulex europaeus agglutinin 1 (UEA-1) (Sigma-Aldrich, St. Louis, MO, United States) and isolated with anti-FITC microbeads (MACS Miltenyi Biotec, San Diego, CA, United States). Cells were then phenotypically defined by flow cytometry as separate biological replicates. Endothelial cell lineage was confirmed by visualizing tube formation with the Cultrex “In Vitro Angiogenesis Assay Tube Formation Kit” (Trevigen, Gaithersburg, MD, United States). UCT-EPC were cultured on gelatin-coated flasks using EGM-2 growth media in a humidified 37°C incubator at 5% CO_2_.

### Culture of Human Hepatic Sinusoidal Endothelial Cells (HHSEC) and Human Umbilical Vein Endothelial Cells (HUVEC)

Human umbilical vein endothelial cells (Lonza, Walkersville, MD, United States) and HHSEC (Science Cell Research Laboratories, Carlsbad, CA, United States) were purchased and expanded in EGM-2 according to manufacturer’s instructions, as previously described in detail (Sanada et al., 2013; Mokhtari et al., 2016).

### Vector Construction, Production, and Purification

The human B-domain deleted fVIII (HSQ) transgene was removed from the HSQ ReNeo plasmid (generously supplied by Dr. Trent Spencer and Dr. Christopher Doering, Department of Pediatrics, Aflac Cancer Center and Blood Disorders, Emory University, Atlanta, GA, United States) by double-digestion with *XhoI* and *NotI* restriction endonucleases (NE Biolabs, Ipswich, MA, United States). The isolated transgene was then ligated into the pLNCX2 retroviral vector plasmid containing an ampicillin resistance gene for bacterial selection and a neomycin phosphotransferase gene for selection of eukaryotic cells following transduction (Takara Bio [formerly Clontech Laboratories], Mountain View, CA, United States). The recombinant plasmids were transformed into Z-Competent *E. coli* cells (Zymo Research, Irvine, CA, United States), and transformed cells were selected by growing bacteria cultures in Terrific Broth (Invitrogen, Carlsbad, CA, United States) supplemented with 100 μg/ml ampicillin (Sigma-Aldrich, St. Louis, MO, United States). Positive clones were confirmed by PCR with primers for the HSQ transgene and by DNA sequencing (Genewiz, South Plainfield, NJ, United States).

Infectious virus was produced by transfection of the RetroPack PT67 retroviral packaging cell line (Takara Bio, Mountain View, CA, United States) using Lipofectamine 3000 (Invitrogen, Carlsbad, CA, United States) according to manufacturer’s instructions. Stable transfectants were selected for 5 days with 500 μg/ml G418 disulfate salt solution (Sigma-Aldrich, St. Louis, MO, United States) starting at 72 h after transfection. Viral supernatants were collected and filtered through 0.45 μm low protein-binding cellulose acetate filters (Pall Corporation, Ann Arbor, MI, United States). The infectious supernatant was then concentrated using a Retrovirus Precipitation Solution (AlStem, Richmond, CA, United States) according to manufacturer’s recommendations. The viral supernatant was stored frozen at −80°C until use, and all further transductions were carried out from the same viral supernatant batch. Infectious titers were determined on NIH/3T3 cells.

### Transduction of Cells With the HSQ Transgene-Encoding Vector

Frozen viral supernatants were thawed and diluted 1:1 in serum-free QBSF-60 media (Quality Biological, Gaithersburg, MD, United States) and 8 μg/ml of protamine sulfate (Calbiochem, San Diego, CA, United States). BM-MSC, UCT-MSC, UCT-EPC, and AF-MSC were transduced at a very low multiplicity of infection (MOI) (approximately, 1 vector to 300 cells, infectious viruses per cell; MOI 0.0032) to ensure single integration events. After overnight incubation at 37°C, cells were cultured in their respective growth medium. Twenty-four hours later, cells underwent antibiotic selection using 350 μg/ml G418 solution (Sigma-Aldrich, St. Louis, MO) for UCT-EPC, or 500 μg/ml G418 disulfate salt solution for BM-MSC, UCT-MSC, and AF-MSC for 5 days, replacing the selection media after 3 days. After antibiotic selection, the remaining transduced cells expressed the neomycin resistance gene (neomycin phosphotransferase II) and the HSQ transgene. Upon confluence, transduced cells were used for supernatant collection, qPCR, and immunofluorescence imaging.

### Reverse Transcription Quantitative PCR (RT-qPCR)

Total RNA was isolated using the RNeasy kit (Qiagen, Valencia, CA, United States) according to manufacturer’s instructions. 200 ng of RNA was converted into cDNA using the RT^2^ First Strand Kit (Qiagen, Valencia, CA, United States). SYBR Green-based qPCR was performed using the qRT-PCR Primer Assay (Qiagen, Valencia, CA, United States) with the following human primers: (1) human fVIII (qRT-F8-FWD: 5′-ATGCACAGCATCAATGGCTAT-3′, qRT-F8-REV: 5′-GTGAGTGTGTCTTCATAGAC-3′) and (2) human vWF (qRT-vWF-FWD: 5′-GGATTCAGTGGATGCAGCAG-3′, qRT-vWF-REV: 5′-TAGGGAGGTCTTCGATTCGC-3′) (Trevisan et al., 2021). Commercially available primers for Human GAPDH were used as an internal reference housekeeping gene (Qiagen, Valencia, CA, United States). The qPCR master mix was loaded into an ABgene Super Plate 96 Well PCR Plate (Thermo Fisher Scientific, Rochester, NY, United States) and processed in the 7300 Real Time PCR System (Applied Biosystems, Grand Island, NY, United States). For each cell type, two biological donors were used and six independent experiments were carried out.

### Immunofluorescence Staining of Cell Cultures

All cell populations were seeded in 2-well glass chamber slides (Thermo Scientific Nunc, Rochester, NY, United States). At 50–80% confluency, staining was performed as previously described (Shahani et al., 2010) with sheep anti-human FVIII:C affinity purified polyclonal antibody (Innovative Research, Novi, MI, United States) and rabbit anti-von Willebrand Factor (vWF) polyclonal antibody (Abcam, Cambridge, MA, United States) or rabbit anti-CD31 polyclonal antibody (Abcam Cambridge, MA, United States). Briefly, slides were fixed, washed, permeabilized, and incubated overnight at 4°C with the primary antibodies diluted in antibody diluent (Dako, Carpinteria, CA, United States). Slides were washed again and incubated with donkey anti-sheep Alexa Fluor 488 and goat anti-rabbit 594 conjugated secondary antibodies for 1 h at room temperature. Slides were washed, counterstained with DAPI (4,6 diamidino-2-phenylindole, Biogenex, Fremont, CA, United States) and mounted with Cytoseal 60 (Thermo Fisher Scientific, Barrington, IL, United States). Immunofluorescence were acquired using a Leica DM4000B Microscope and a 40× objective (Leica, Buffalo Grove, IL, United States). One well stained with only secondary antibody served as a negative control in all slides tested. Following data acquisition, minimal global processing of images was performed with Adobe Photoshop CS6.

### Flow Cytometry Analysis of BiP and CHOP Protein Expression

This assay included UCT-EPC, UCT-MSC, AF-MSC, BM-MSC, transduced and non-transduced, and same passage UCT-EPC, UCT-MSC, AF-MSC, BM-MSC, treated with tunicamycin (Cell signal Technology, Danvers, MA, United States) either overnight at a concentration of 1 ug/mL (BiP positive control), or for 8 h at a concentration 2 ug/mL (CHOP positive control) as previously described (El-Akabawy et al., 2020). Cells were harvested from culture, washed, fixed with 4% paraformaldehyde, and permeabilized with methanol using the intracellular flow cytometry kit (Cell Signal Technology, Danvers, MA, United States) and the protocol provided by the manufacturer. Samples were washed with PBS and were then stained with rabbit anti-human BiP conjugated with Phycoerythrin, or mouse anti-human CHOP conjugated with Alexa Fluor^®^ 647, and respective concentration-matched isotype controls according to manufacturer’s instructions. Labeled cells were run and analyzed separately using a BD Accuri C6 Plus (BD Biosciences, San Jose, CA, United States) and FlowJo software (version 10.6.1; Tree Star, Ashland, OR, United States).

### Activated Partial Thromboplastin Time (aPTT) Assays to Measure FVIII

At about 70% confluence, all cell populations (that were plated at the same initial density) were cultured in serum-free QBSF-60 media (Quality Biological, Gaithersburg, MD, United States) for 48 h. Supernatants were collected at 48 h and the total number of cells was counted. The media was centrifuged at 1500 RPM for 5 min at 4°C to remove cellular debris, and the remaining supernatant was collected and stored frozen at −80°C until use. Aliquots of each frozen supernatant were brought to the Wake Forest Baptist Medical Center Special Hematology Laboratory, where FVIII:C activity was measured by aPTT clotting time using the facility’s clinical coagulometer, appropriately adjusting the standards to enable accurate quantitation of the low levels of FVIII present in the culture supernatants.

### Statistical Analysis

Experiments were independently repeated at least three times and results are presented as mean ± SD. Student’s *t*-test was used to analyze the statistical significance of the results, and *p* values < 0.05 were considered to be statistically significant.

## Results

### Isolation and Characterization of Cells From Prenatal, Neonatal, and Adult Tissues

**FIGURE 1:**
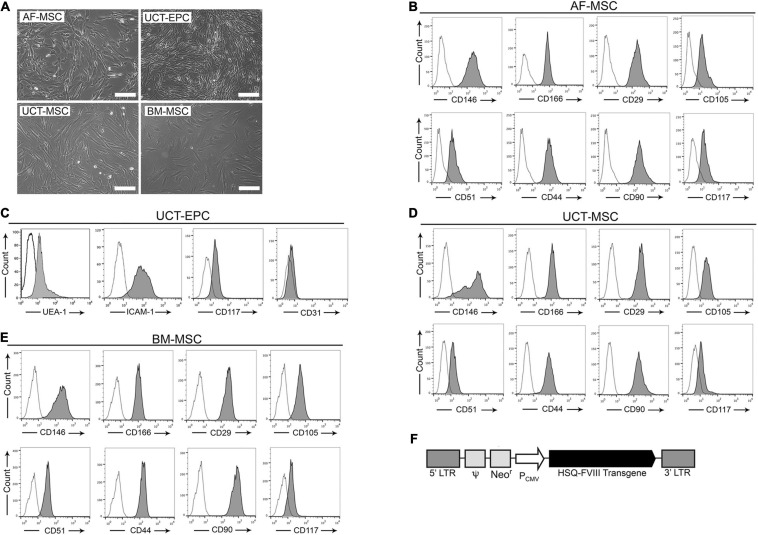
Isolated cells were characterized via light microscopy and flow cytometry. **(A)** Cells visualized microscopically all had spindle-shaped morphology. Scale bar = 200 μm. Flow cytometric analysis was performed on isolated: **(B)** amniotic fluid-derived mesenchymal stromal cells (AF-MSC), **(C)** cord tissue-derived endothelial progenitor cells (UCT-EPC), **(D)** cord tissue-derived mesenchymal stromal cells (UCT-MSC), and **(E)** bone marrow-derived mesenchymal stromal cells (BM-MSC). **(F)** Schematic representation of the MMLV-based vector encoding the B domain-deleted human fVIII transgene (HSQ) under the control of the CMV promoter.

The amniocentesis specimens collected for prenatal genetic screenings contain a population of cells expressing c-Kit (CD117) that display mesenchymal stromal cell (MSC) markers and can be isolated and grown in culture as stable lines (De Coppi et al., 2007). These amniotic fluid MSC (AF-MSC) are thus an accessible, autologous source for prenatal cell-based therapies (Troeger et al., 2006; Ishii and Eto, 2014). As shown in Figure 1A, AF-MSC (De Coppi et al., 2007) exhibited a spindle-shaped morphology, and flow cytometry confirmed they expressed multiple MSC markers (Figure 1B) and were devoid of hematopoietic (CD45, CD34) and endothelial (CD31) markers (data not shown).

If amniocentesis cannot be performed, alternative tissues can be obtained neonatally. Umbilical cord tissue derived- MSC (UCT-MSC) and endothelial progenitor cells (UCT-EPC) can be isolated at birth in relatively large numbers, providing another accessible, autologous source for cellular therapies (Ingram et al., 2004; Maleki et al., 2014). Umbilical cord tissue (UCT) from two human donors was used to isolate UCT-MSC and UCT-EPC (detailed in Materials and Methods), both of which adhered within 24 h after plating and exhibited a spindle-shaped morphology (Figure 1A). Flow cytometry demonstrated that UCT-EPC expressed UEA-1, CD117, and low levels of VEGFR2/CD309 and CD31 (Figure 1C), while UCT-MSC expressed several established MSC markers (Figure 1D).

**FIGURE 2:**
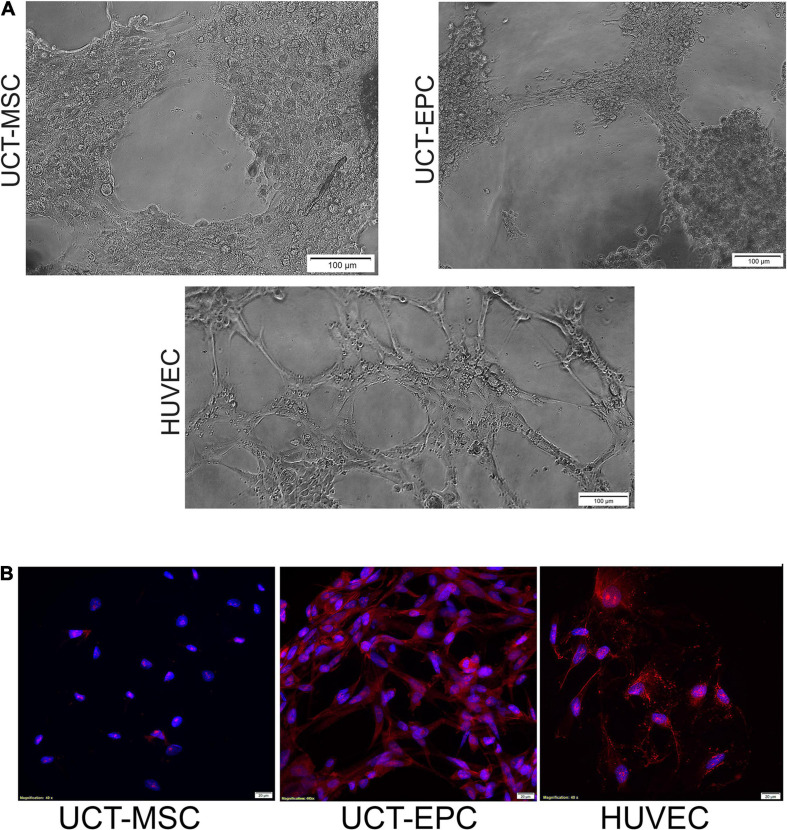
**(A)** Functionality of UCT-MSC and UCT-EPC was determined by tube-forming assays and compared to HUVEC as a positive control. While quantification of number of branch sites/nodes demonstrated UCT-EPC tube-formation was not as robust as that of HUVEC, UCT-MSC did not form any tube structures. Cells initiated tubule formation at 4 h of culture, which fully developed by 6 h, with the presence of nodes of ≥4 branches; images were acquired on a Ziess Axiovert 200M and 10× magnification. **(B)** Immunostaining of UCT-MSC and UCT-EPC in culture, demonstrated that the latter were positive for CD31. Images were acquired on a Ziess Axiovert 200M and 40× magnification.

Functionality of isolated UCT-EPC was confirmed by tube-forming assays and compared to UCT-MSC, using HUVEC as a positive control (Figure 2A). While UCT-EPC tube-formation was not as robust as that of HUVEC, UCT-MSC did not form tube structures when plated in this assay (Figure 2A). In addition, immunostaining of UCT-MSC and UCT-EPC in culture demonstrated that the latter were positive for CD31 (Figure 2B).

While autologous fetal tissue cannot be isolated to treat adult patients, bone marrow-derived MSC (BM-MSC) serve as an alternate vehicle for FVIII delivery (Porada et al., 2011; Sanada et al., 2013). BM-MSC also had a spindle shape (Figure 1A), expressed characteristic MSC markers (Figure 1E), were devoid of hematopoietic markers, and differentiated into osteocytes, chondrocytes, and adipocytes in appropriate media (Soland et al., 2012).

Population doublings per passage were also determined for the different cell populations. Although all cells were used between passage 3 and 7, BM-MSC had undergone 2.5–3 population doublings per passage, AF-MSC 4 population doublings per passage, while UCT-MSC and UCT-EPC both underwent 5–5.5 population doublings per passage.

### FVIII mRNA Expression in AF-MSC, UCT-EPC, UCT-MSC, and BM-MSC

**FIGURE 3:**
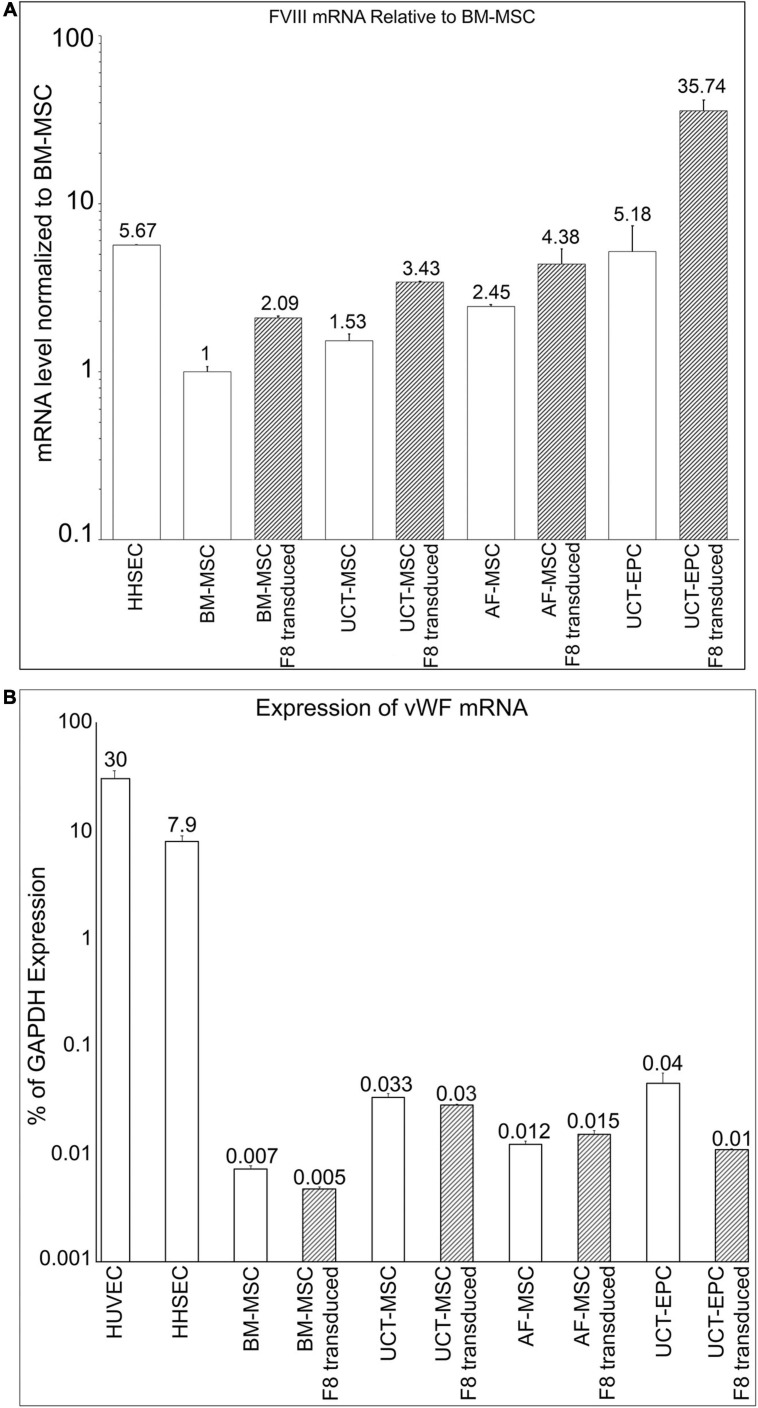
**(A)** Quantification of FVIII mRNA in the different cell populations prior to (open bars) and following (hatched bars) transduction with an HSQ-encoding MMLV vector was performed by RT-qPCR. Human hepatic sinusoidal endothelial cells (HHSEC) were used as a positive control. The level of FVIII mRNA in untransduced BM-MSC was set equal to “1” and the level of FVIII mRNA in each of the other samples is expressed as fold-difference relative to that value. Values represent the average of two different donors (biological replicates) for each cell type and 6 independent experiments. Error bars represent standard error of the mean (SEM). **(B)** Quantification of vWF mRNA in the different cell populations prior to (open bars) and following (hatched bars) transduction with an HSQ-encoding MMLV vector was performed by RT-qPCR. Human hepatic sinusoidal endothelial cells (HHSEC) and HUVEC were used as positive controls. The levels of vWF mRNA are expressed as a percentage of each sample’s respective level of GAPDH mRNA. Values represent the average of two different donors (biological replicates) for each cell type and 6 independent experiments. Error bars represent standard error of the mean (SEM).

Next, we quantified the levels of FVIII mRNA in these four cell types with RT-qPCR. Following baseline mRNA quantification, cells were transduced with an MMLV-based vector encoding a B domain-deleted human fVIII transgene (HSQ) (Figure 1F), as detailed in Materials and Methods. After expanding the transduced cells, we repeated FVIII mRNA quantitation with RT-qPCR. As human hepatic sinusoidal endothelial cells (HHSEC) are believed to be responsible for FVIII production *in vivo* (Fahs et al., 2014; Shahani et al., 2014), they served as a positive control. All cell types were then compared to human BM-MSC, whose endogenous FVIII expression level was set to 1. Compared to BM-MSC, all other cell types exhibited significantly higher basal FVIII mRNA levels. Specifically, UCT-MSC and AF-MSC exhibited 53 and 145%, respectively, higher levels of FVIII mRNA than BM-MSC, while UCT-EPC endogenously produced 418% more FVIII mRNA than BM-MSC, placing them nearly on par with HHSEC (467% of BM-MSC). These data (*n* = 6) appear in the open/white bars in Figure 3A.

Following transduction, all cell types exhibited markedly enhanced FVIII mRNA levels, with BM-MSC, UCT-MSC, and AF-MSC each doubling their FVIII mRNA levels. UCT-EPC demonstrated a remarkable ability to express FVIII upon transduction, producing levels of FVIII mRNA roughly 7-times that of HHSEC. These data (*n* = 6) appear in the hatched bars in Figure 3A.

### vWF mRNA Expression

Upon synthesis (or infusion), FVIII almost immediately binds non-covalently to its carrier protein, vWF. Within HHSEC and other endothelial cells that serve as natural sites of FVIII synthesis within the body, FVIII complexes with vWF as it is synthesized, and these complexes are stored in cytoplasmic Weibel-Palade bodies (WPB) (Shahani et al., 2014). Complexing to vWF protects FVIII from rapid proteolytic degradation by circulating phospholipid-binding proteases (Lenting et al., 2007) and is also thought to decrease its perceived immunogenicity (Dasgupta et al., 2007; Herczenik et al., 2012). We, therefore, next investigated endogenous expression of vWF mRNA by MSC and EPC from BM, AF, and UCT, and asked whether transduction of these cells with an HSQ-encoding MMLV vector would upregulate vWF to match the increased FVIII production (*n* = 6). HUVEC and HHSEC both endogenously produce vWF and were used as positive controls. RT-qPCR confirmed that both HUVEC and HHSEC produced very high levels of vWF mRNA. Somewhat surprisingly, all other cell types also endogenously expressed vWF mRNA, albeit at very low levels when expressed as a percent of mRNA for the housekeeping gene GAPDH (Figure 3B, open bars). UCT-MSC and UCT-EPC exhibited the highest vWF mRNA levels (5–6 times that of BM-MSC), while AF-MSC expressed levels of vWF mRNA that were intermediate between those of BM-MSC and those of the cord tissue-derived cells (Figure 3B, open bars). Interestingly, over-expressing HSQ (FVIII) via transduction did not increase vWF mRNA levels in any of the cells, and actually significant decreased vWF mRNA levels in the UCT-EPC, the cells with the highest endogenous vWF mRNA levels.

### FVIII Is Not Stored in Granules/WPB

**FIGURE 4:**
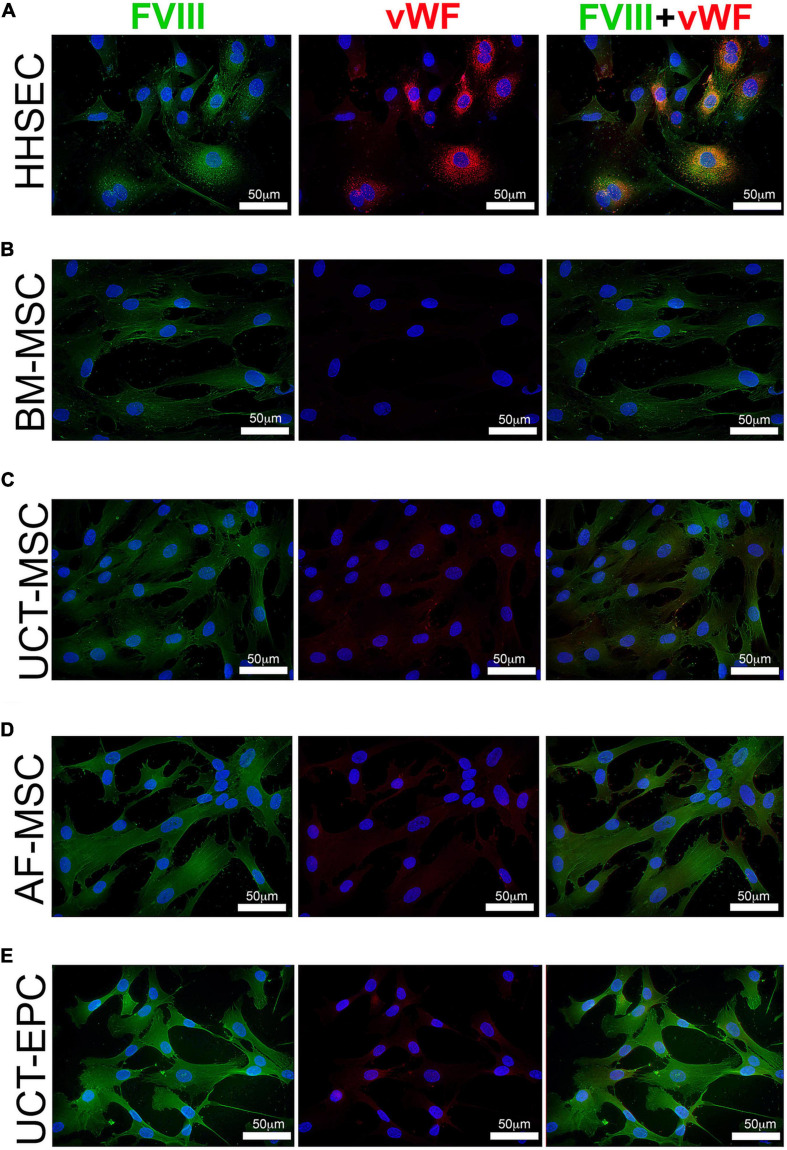
Immunofluorescence visualization of FVIII and vWF expression. Cells were grown in chamber slides, fixed, and stained with antibodies to FVIII (green) and vWF (red), while nuclei were counterstained with DAPI (blue). **(A)** HHSEC (positive control) demonstrate high level expression of both FVIII and vWF, and these two proteins colocalize and exhibit a punctate staining pattern consistent with storage within Weibel-Palade bodies (WPB). In contrast, while expression of MMLV-encoded HSQ (FVIII; green) can readily be detected in all transduced cell types, no vWF (red) staining is seen in BM-MSC **(B)**, and only very low levels of staining for vWF (red) are seen in UCT-MSC **(C)**, AF-MSC **(D)**, and UCT-EPC **(E)**. Moreover, staining for the exogenous MMLV-encoded FVIII (green) is diffusely spread throughout the cytoplasm, suggesting that it is not being stored within WPB as it is in HHSEC.

Since all of the cell types endogenously expressed very low levels of vWF, we next examined whether the MMLV-derived FVIII was complexed with vWF, packaged, and stored in granules/WPB as occurs in sites of native FVIII synthesis. To answer this question, we performed immunofluorescence with antibodies to vWF and FVIII on transduced cells, looking for colocalization of these signals and for the presence of punctate staining indicative of protein storage in granules. As HHSEC produce and store FVIII:vWF complexes in WPB, they were a positive control (Figure 4A). As shown in Figures 4B–E, transduced BM-MSC, UCT-MSC, AF-MSC, and UCT-EPC all expressed readily detectable amounts of FVIII protein (green). UCT-EPC expressed the most protein, in agreement with the preceding mRNA quantitation. Despite the presence of low levels of vWF mRNA in all of these cell types, however, only a very faint signal was present (if at all) in any of these cells following staining with an antibody to vWF (red). Moreover, in HHSEC, bright, punctate, colocalized staining for both FVIII (green) and vWF (red) was observed, confirming the formation of FVIII:vWF complexes stored within WPB. In contrast, in the transduced cells, FVIII protein was not stored in granules/WPB, but instead was diffusely cytoplasmic/perinuclear. These findings agree with our prior studies demonstrating endogenous low-level expression of FVIII in BM-MSC (Sanada et al., 2013). Images were acquired in each channel (color) using identical camera settings from cell type to cell type, to enable fluorescence intensity to serve as a readout for relative protein levels.

### Activity of Secreted FVIII

**FIGURE 5:**
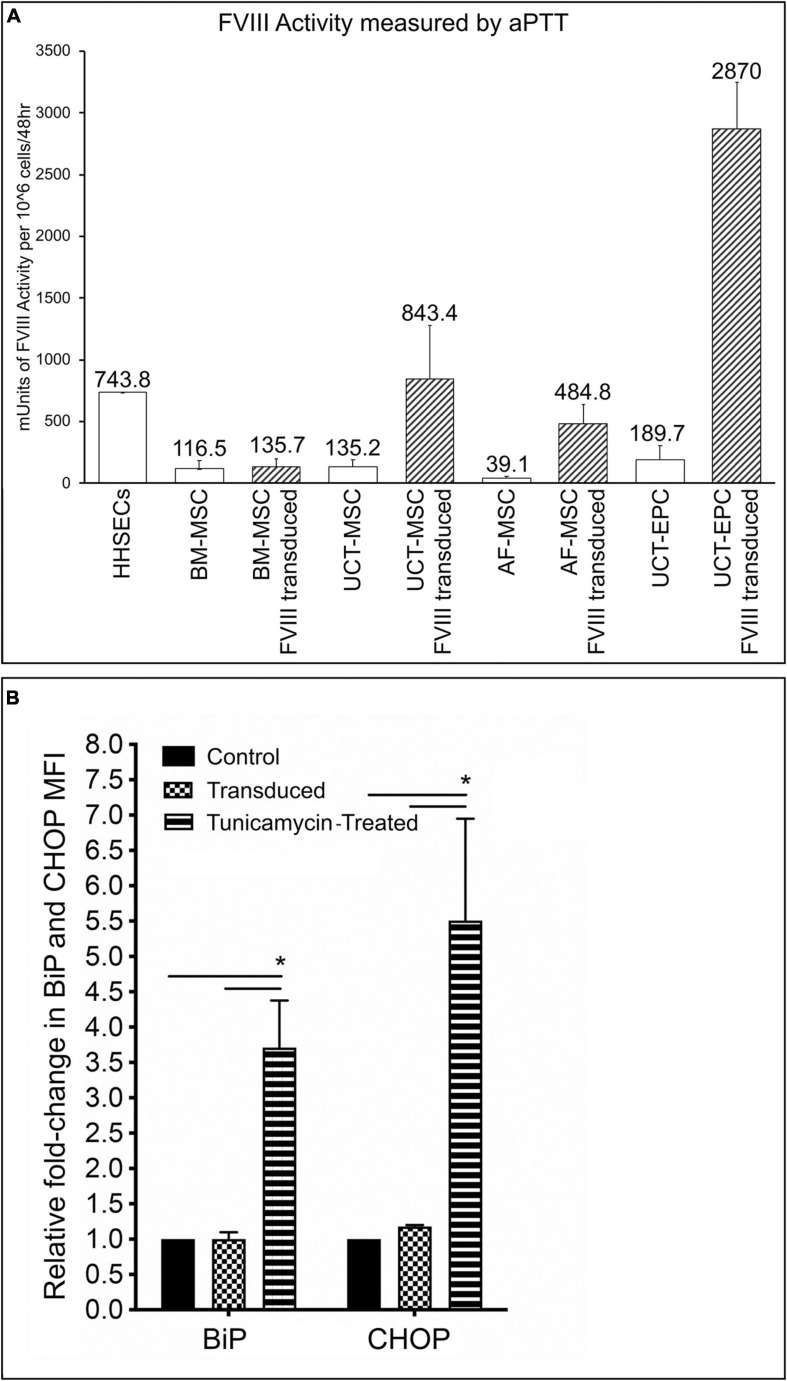
**(A)** Measurement of FVIII:C activity by aPTT assay. 48-h supernatants were collected from each of the cell types, prior to and following transduction with the HSQ-encoding MMLV vector, and FVIII activity was measured by activated partial thromboplastin time (aPTT) assay. Values are expressed in mUnits of FVIII:C activity per million cells per 48 h. Error bars represent standard error of the mean (SEM). **(B)** Flow cytometric analysis of BiP and CHOP, in untransduced, transduced cells, and cells treated with tunicamycin (positive control). Fold-change in MFI of BiP and CHOP, was calculated by dividing the MFI of BiP or CHOP in transduced, or tunicamycin-treated cells, by the MFI of non-transduced cells, after subtracting respective isotype controls. ^∗^*p* < 0.05.

While expression of FVIII at the level of mRNA and protein are important from the standpoint of identifying a promising cellular vehicle to deliver a fVIII transgene to treat HA, what is needed to correct the disease phenotype is procoagulant (FVIII:C) activity. We therefore used aPTT assays to examine the levels of FVIII:C activity in culture supernatants from each cell type, both prior to and following transduction with the HSQ-encoding MMLV vector, as detailed in the Materials and Methods. As shown in Figure 5A, all cell types expressed detectable FVIII:C activity prior to transduction (open bars) that was well below that of HHSEC (positive control). Following transduction (hatched bars), FVIII:C activity rose markedly in UCT-MSC and AF-MSC to levels comparable to that of HHSEC, while the levels in transduced UCT-EPC were roughly 4-times those of the HHSEC, results which correlated well with the preceding results with mRNA and protein (immunofluorescence) levels. However, and in with our previous report using BM-derived MSC from sheep (Porada et al., 2011), transduction of BM-MSC only led to a modest increase in the levels of FVIII:C being secreted into the supernatant, which did not correlate with the doubling seen at the mRNA level and the marked increase in immunofluorescence staining intensity. Since the extracellular association of FVIII with vWF may be necessary for efficient secretion of FVIII from its cell of origin (Lollar, 1991) and BM-MSC do not produce vWF, it is likely that the lack of vWF in the supernatant impacted the levels of FVIII detected by aPTT.

Another possible explanation would be that in BM-MSC a mis-folded or mis-modified FVIII protein could not be processed in the endoplasmic reticulum (ER). Thus, we next investigated whether transduction of BM-MSC led to upregulation of the UPR sentinel chaperone BiP (binding immunoglobulin protein), since its synthesis was reported to be significantly induced by accumulation of misfolded FVIII proteins in the ER (Brown et al., 2011; Lange et al., 2016; Zolotukhin et al., 2016). In addition, we also examined whether the downstream signaling, proapoptotic protein C/EBP homologous protein (CHOP) was upregulated. In addition, we also evaluated BiP and CHOP expression in UCT-MSC, UCT-EPC, and AF-MSC. No statistically significant differences were found between BiP and CHOP expression in BM-MSC, UCT-MSC, UCT-EPC, or AF-MSC before and after transduction. The results of flow cytometric analysis of BiP and CHOP for the 4 cell types, grouped together, are shown in Figure 5B. Expression of BiP and CHOP before (BiP: 1.1 ± 0.1; CHOP: 1.2 ± 0.02) and after stimulation with tunicamycin (BiP: 4.1 ± 0.61; CHOP: 6.8 ± 1.8) (positive control). In addition, no significant upregulation of BiP or CHOP was seen in any of the 4 cell types overexpressing FVIII when compared to their baseline, as determined by calculating the fold-change in mean fluorescent intensity (MFI) of BiP and CHOP, after subtracting respective isotype controls (Figure 5B).

## Discussion

Herein, we identified several fetal and postnatal cell types that are readily available and could potentially serve as effective cellular vehicles for delivering a fVIII transgene. Depending upon the cell source, these could represent an autologous product to treat HA, prior to or after birth. Given their ease of isolation, their ability to be expanded in culture, their suitability as gene delivery vehicles, and the observation that they endogenously produce functional FVIII (Wood et al., 2007, 2008; Porada and Almeida-Porada, 2010; Sanada et al., 2013), we focused on EPC and MSC from several sources of varying ontogenic stages. Since they are the only autologous cell source that could be obtained and re-administered prenatally, we initially focused on AF-MSC. These cells performed far better than MSC derived from adult BM with respect to FVIII production levels, both prior to and following transduction with an HSQ-encoding MMLV-based vector. Combined with their demonstrated ability to engraft and persist long-term in multiple tissues after prenatal delivery (Shaw et al., 2011b, 2016; Yang et al., 2013; Subramaniam et al., 2018), AF-MSC could represent a viable autologous cellular platform for delivering a FVIII transgene *in utero*. However, the use of autologous gene-modified AF-MSC prenatally would not be trivial, given the length of time required for their transduction and subsequent *in vitro* expansion, and the limited temporal window during which *in utero* transplantation can be performed and immune tolerance to the exogenous transgene product induced (Tran et al., 2001; Waddington et al., 2003; Porada et al., 2004; Colletti et al., 2008). As such, allogeneic “off-the-shelf” cells may be more practical for rapid *in utero* intervention.

Our studies demonstrate that cells derived from umbilical cord tissue (UCT-MSC and UCT-EPC) yield the highest levels of FVIII mRNA, protein, and FVIII:C activity following transduction with an HSQ-encoding MMLV vector. In particular, UCT-EPC proved to be highly amenable to expressing vector-encoded FVIII, producing levels of mRNA, protein, and procoagulant activity that far exceeded that of HHSEC, cells thought to be the body’s primary site of FVIII synthesis (Fahs et al., 2014). While not quite as robust as UCT-EPC, upon transduction, UCT-MSC produced levels of FVIII and FVIII:C activity comparable to those of HHSEC. Since UC is a discarded tissue, it is readily available, and its use has no ethical ramifications. In addition, the high proliferative potential and rapid doubling times ensure that clinically relevant numbers of UCT-derived cells, i.e., a total of 10^7^ (clinically employed dose: 10^7^–10^8^ cells/kg fetal weight) could quickly be obtained. In similarity to AF-MSC, *in utero* transplantation of MSC and EPC results in long term engraftment in treated animals without any adverse effects (Colletti et al., 2009; Wood et al., 2012; Mokhtari et al., 2016). Thus, these qualities combine to make UCT-MSC and UCT-EPC particularly attractive candidates for cellular delivery vehicles for FVIII, and likely other gene products as well.

Our finding that all cell types tested expressed vWF mRNA (and in some cases protein), albeit at very low levels, is surprising, since vWF expression is generally accepted to be restricted to the endothelial lineage (Crisan et al., 2012). However, recent studies have suggested a close, perhaps fluid, developmental relationship between the MSC and EPC lineages, (Shafiee et al., 2015, 2018). Indeed, our immunophenotyping revealed a high degree of similarity/overlap between the UCT-MSC and UCT-EPC. Yet, only the UCT-EPC isolated based on expression of UEA-1 were able to form tubule networks, confirming their endothelial functionality, and thus distinguishing them from the phenotypically similar UCT-MSC. It is interesting to note that UCT-MSC and UCT-EPC were the cell types that exhibited the highest levels of endogenous vWF mRNA, suggesting a possible link between their expression of vWF and their ability to express very high levels of FVIII following transduction.

## Conclusion

Our findings suggest that all cell types we investigated represent viable candidates for use as cellular vehicles to deliver a fVIII transgene to treat HA. Nonetheless, the marked differences in the levels of FVIII produced by similar cell types isolated from different tissues pave the way for future studies to define the mechanistic basis for these differences and develop means of harnessing the responsible pathways to drive high-level FVIII expression in other clinically valuable cell types. In addition, it is likely the use of more advanced vector systems, a bioengineered fVIII transgene (Doering et al., 2004, 2009; Brown et al., 2014, 2018), and/or performing cell-specific codon-optimization of the fVIII transgene could further improve FVIII production and secretion; these are all areas we are actively pursuing.

## Data Availability Statement

The raw data supporting the conclusions of this article will be made available by the authors, without undue reservation, to any qualified researcher.

## Ethics Statement

The studies involving human participants were reviewed and approved by Office of Human Research Protection at Wake Forest Health Sciences. The patients/participants provided their written informed consent to participate in this study.

## Author Contributions

CS, CR, RR, and SG performed experiments, data analysis, and interpretation. DM and AF provided expertise. CD, HS, and AA provided reagents, materials, and experimental feedback. GA-P, CP, CD, and HS approved final version of the manuscript. CR provided manuscript draft. GA-P and CD contributed to conception and experimental design, supervised experiments, performed data analysis and interpretation, wrote the manuscript, and secured funding. All authors contributed to the article and approved the submitted version.

## Conflict of Interest

The authors declare that the research was conducted in the absence of any commercial or financial relationships that could be construed as a potential conflict of interest.

## Publisher’s Note

All claims expressed in this article are solely those of the authors and do not necessarily represent those of their affiliated organizations, or those of the publisher, the editors and the reviewers. Any product that may be evaluated in this article, or claim that may be made by its manufacturer, is not guaranteed or endorsed by the publisher.
